# Effects of *Alexandrium pacificum* Exposure on *Exopalaemon carinicauda*: Hepatopancreas Histology, Antioxidant Enzyme Activity, and Transcriptome Analysis

**DOI:** 10.3390/ijms26041605

**Published:** 2025-02-13

**Authors:** Wanyu Han, Weitao Cheng, Menghao Fan, Dexue Liu, Yanrong Cao, Xuao Mei, Jiaxuan Wan, Guangwei Hu, Huan Gao, Nanjing Ji

**Affiliations:** 1 Jiangsu Key Laboratory of Marine Bioresources and Environment, Jiangsu Ocean University, Lianyungang 222005, China; hwy13604327758@163.com (W.H.); cwt18109635480@163.com (W.C.); mh1790735695@163.com (M.F.); xue1553906987@163.com (D.L.); cyr520791214@163.com (Y.C.); 17670571813@163.com (X.M.); 17851971214@163.com (J.W.); huanmr@163.com (H.G.); 2 Co-Innovation Center of Jiangsu Marine Bio-industry Technology, Jiangsu Ocean University, Lianyungang 222005, China; 3 The Jiangsu Provincial Infrastructure for Conservation and Utilization of Agricultural Germplasm, Nanjing 210014, China

**Keywords:** *Exopalaemon carinicauda*, *Alexandrium pacificum*, transcriptomic, hepatopancreas damage, harmful algal blooms

## Abstract

*Alexandrium pacificum*, a dinoflagellate known for causing harmful algal blooms (HABs), has garnered significant attention due to its potential toxicity to marine ecosystems, fisheries, and human health. However, the effects of this toxin-producing alga on shrimp are not yet comprehensively understood. This study aimed to assess the hepatopancreas damage induced by *A. pacificum* in the economically important shrimp species *E. carinicauda* and to elucidate the underlying molecular mechanisms through histology, antioxidant enzyme activity, and transcriptome analysis. The shrimp were assigned to either a control group or an exposed group, with the latter involving exposure to *A. pacificum* at a concentration of 1.0 × 10^4^ cells/mL for 7 days. A histological analysis subsequently revealed pathological changes in the hepatopancreas tissue of the exposed group, including lumen expansion and the separation of the basement membrane from epithelial cells, while antioxidant enzyme activity assays demonstrated that exposure to *A. pacificum* weakened the antioxidant defense system, as evidenced by the reduced activities of catalase, superoxide dismutase, and glutathione, along with increased malondialdehyde levels. Transcriptome analysis further identified 663 significantly upregulated genes and 1735 significantly downregulated ones in the exposed group, with these differentially expressed genes being primarily associated with pathways such as protein processing in the endoplasmic reticulum, mitophagy, glycolysis/gluconeogenesis, sphingolipid metabolism, and glycerophospholipid metabolism. This study provides novel insights into the toxicological effects of *A. pacificum* on aquatic organisms and enhances the current understanding of the ecotoxicological risks posed by HABs.

## 1. Introduction

*Exopalaemon carinicauda*, a member of the suborder *Pleocyemata* and family *Palaemonidae*, is an economically significant pond-reared shrimp species in China [1,2]. Indeed, it is highly valued in the aquaculture industry due to its rapid growth, short breeding cycle, good adaptability, and long reproductive season which have led to a significant expansion in the species’ breeding area [3,4]. However, the growing scale of shrimp farming has also brought challenges, including threats from vibrio [5,6], viruses [7,8], and even dinoflagellates [9,10]. Dinoflagellates are single-celled microeukaryotes, of which approximately half have photosynthetic abilities, thereby contributing significantly to the primary productivity of aquatic ecosystems [11]. However, the rapid proliferation of certain dinoflagellate species can also lead to harmful algal blooms (HABs) that negatively impact both aquatic environments and aquaculture operations [12]. HABs have been reported in shrimp ponds across various regions worldwide [13,14], and different studies have highlighted the negative impact of harmful dinoflagellates on shrimp mobility, survival, oxidative stress responses, tissue health, growth, and disease resistance [10,15,16], with these effects ultimately leading to significant economic losses for the aquaculture industry.

The armored dinoflagellate genus *Alexandrium* is one of the most widespread HAB species [15]. Previous research indicated that *Alexandrium tamarense* can adversely affect the early developmental stages of scallops, resulting in developmental abnormalities and reduced survival rates [16]. Furthermore, toxic algae have been found to exert significant lethal and sub-lethal effects on fish larvae, whether in isolation or in mixtures [17]. In particular, exposure to sub-lethal levels of the toxic dinoflagellate *Alexandrium fundyense* was shown to significantly reduce reproduction and hatching success in the copepod *Calanus finmarchicus*, although it had minimal effects on survival rates and feeding behavior [18]. Similarly, studies revealed that the toxic dinoflagellate *Alexandrium catenella* could disrupt energy metabolism, alter the composition of osmotic pressure-regulating substances in blood cells, and modify lipid profiles in the blue mussel *Mytilus edulis* [19]. Collectively, these findings highlight the significant impact of harmful dinoflagellates on aquatic organisms. *A. pacificum* is a notable dinoflagellate species recognized for its harmful effects, particularly its ability to produce paralytic shellfish toxins [20]. However, its negative impact on cultured shrimp, especially *E. carinicauda*, remains poorly understood. It is, therefore, crucial to investigate the effects of *A. pacificum* on *E. carinicauda* in order to gain a comprehensive understanding of how harmful microalgae affect shrimp and to develop strategies for mitigating these risks in aquaculture systems.

The hepatopancreas is the primary organ responsible for nutrient absorption, metabolism, digestion, and storage in decapod crustaceans. It also plays a crucial role in energy metabolism under environmental stress [21,22]. Hence, the dysfunction of the hepatopancreas can result in metabolic disorders, immunosuppression, and even mortality [23]. Consequently, the hepatopancreas serves as an optimal tissue for examining the impacts of environmental stressors on shrimp. However, the specific effects of harmful algal exposure on the hepatopancreas of *E. carinicauda* have not yet been elucidated. This study, therefore, concentrated on the hepatopancreas as the focal organ to assess the impact of *A. pacificum* exposure on *E. carinicauda*. Notably, *E. carinicauda* is both an important species in aquaculture and a model organism for investigating the physiological responses of crustaceans to environmental stressors. [24,25]. The main aim of this study was to examine *A. pacificum*-induced hepatopancreas damage in *E. carinicauda*. The findings of the current study provide novel insights into the toxicity mechanisms of harmful toxin-producing dinoflagellates in aquatic animals and contribute to an ecological risk assessment of harmful algal blooms in aquaculture systems.

## 2. Results

### 2.1. Histological Assays

**Figure 1:**
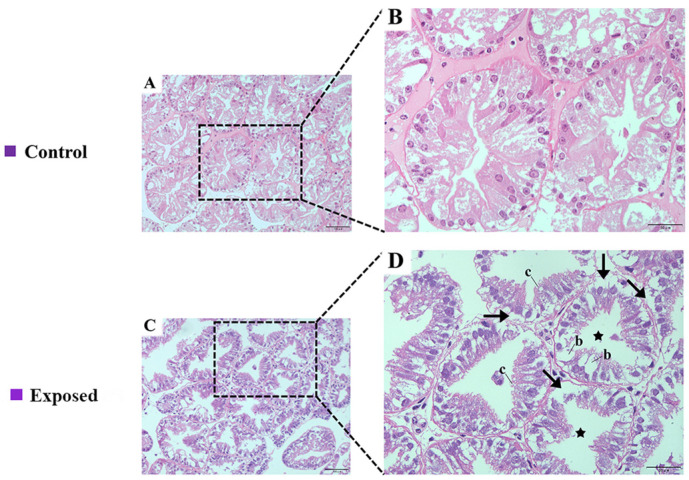
Histopathological effects of *A. pacificum* exposure on the hepatopancreas of *E. carinicauda*. Control group (**A**,**B**); exposed group (**C**,**D**). Note: (**A**,**C**) 200 × magnification; (**B**,**D**) 400× magnification. The part circled in the black box is enlarged. Black arrow: separation of the basement membrane from epithelial cells and cell shedding; black star: lumen dilatation; b: B cells; c: R cells.

Significant histological alterations were observed in the hepatopancreas of *E. carinicauda* following exposure to *A. pacificum*. In the control group, the hepatopancreas exhibited well-organized, rounded glandular tubes with an intact cellular morphology. The glandular ducts were also tightly arranged, while the lumen displayed a stellate structure (Figure 1A,B). In the exposed group, after exposing the shrimp to *A. pacificum* for 7 days, the hepatopancreas showed increased numbers and sizes of B cells, lumen dilation, a higher abundance of R cells, and the separation of the basement membrane from the epithelial layer (Figure 1C,D).

### 2.2. Antioxidant Parameters

**Figure 2:**
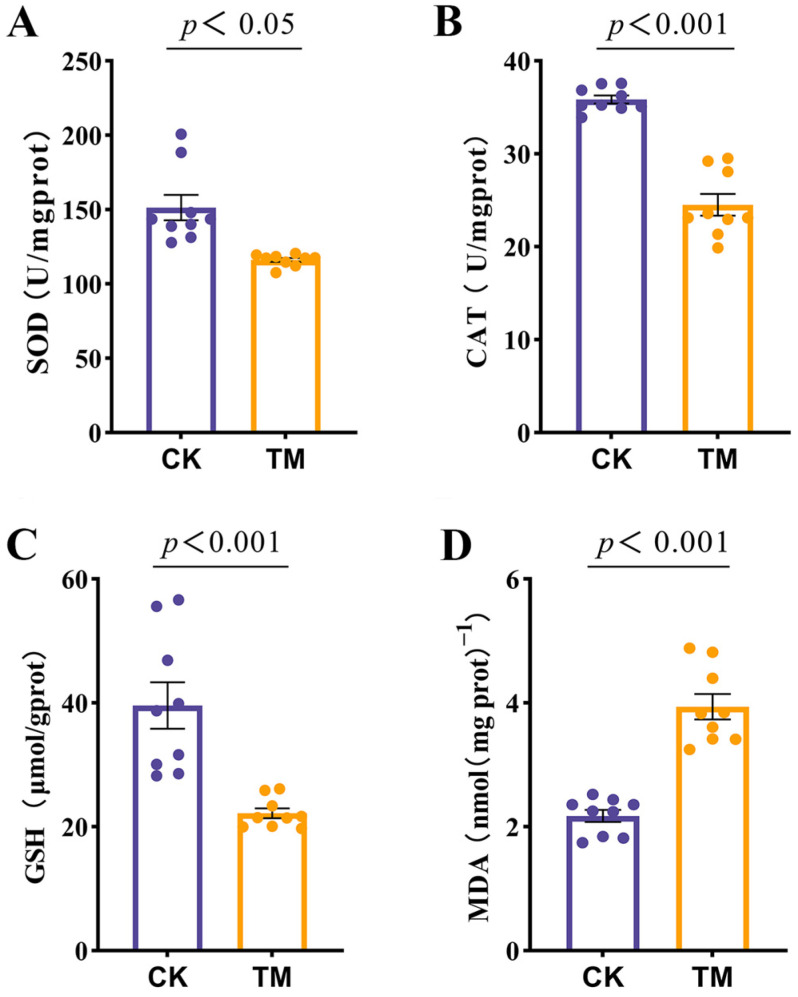
Effects of *A. pacificum* on antioxidant parameter in the hepatopancreas of *E. carinicauda*. CK: control group; TM: exposed group; (**A**) Superoxide dismutase (SOD) activity; (**B**) Catalase (CAT) activity; (**C**) Glutathione (GSH) content; (**D**) Malondialdehyde (MDA) content.

In the exposed group, *A. pacificum* significantly decreased the levels of glutathione (GSH), catalase (CAT), and superoxide dismutase (SOD) in *E. carinicauda*, while the malondialdehyde (MDA) content showed a significant increase compared with the control group (*p* < 0.05; Figure 2A–D). These results indicated that *A. pacificum* exposure can induce oxidative stress, leading to the depletion of antioxidant defenses and enhanced lipid peroxidation in *E. carinicauda*.

### 2.3. DEGs in the Hepatopancreas After A. pacificum Exposure

**Figure 3:**
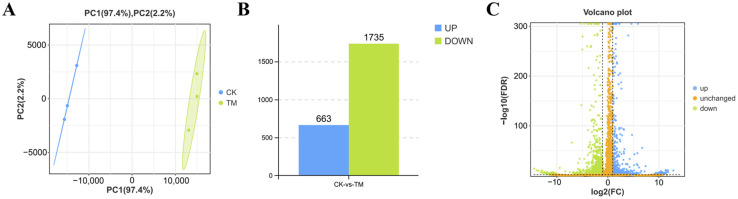
DEGs between the hepatopancreas of *E. carinicauda* from the exposed and control groups. (**A**) Correlations between the exposure and control samples. (**B**) Number of significantly upregulated and downregulated genes in the two groups. (**C**) Volcano plot highlighting the upregulated and downregulated genes identified via RNA-seq. CK: control group; TM: exposed group.

Transcriptome sequencing generated clean reads ranging from 6,322,223,082 to 7,749,082,188 bp, with Q20 and Q30 values reaching 98.26% and 94.78%, respectively. In addition, the GC content ranged from 44.64% to 45.31% (Table S1), hence confirming the reliability of the transcriptome data. PCA further revealed a distinct separation between the samples from the exposed and control groups (Figure 3A), indicating significant differences in the gene expression patterns of their hepatopancreas tissues after being exposed to *A. pacificum*. Finally, 2398 DEGs, including 663 upregulated and 1735 downregulated genes, were identified in the hepatopancreas (Figure 3B,C). Raw data were uploaded to the National Center for Biotechnology Information (NCBI) (accession number is PRJNA1212343).

### 2.4. GO Enrichment Analysis

**Figure 4:**
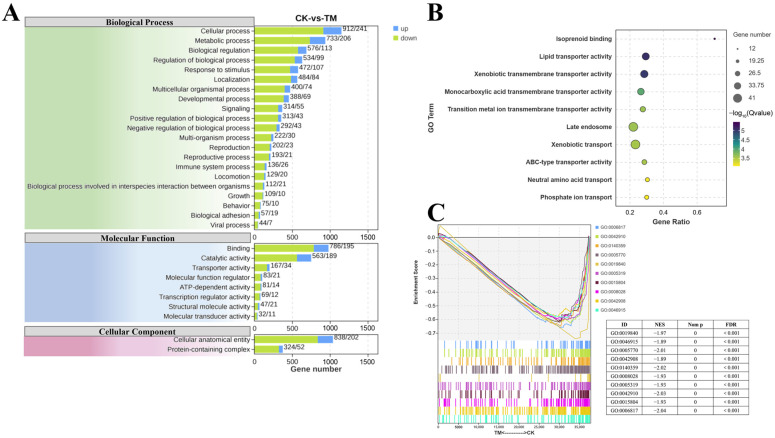
GO enrichment analysis of DEGs in the hepatopancreas of *E. carinicauda* from the exposed and control groups. (**A**) GO terms at level 1 and level 2. (**B**) Differential GO terms associated with protein trafficking and metabolism. (**C**) The GSEA results for protein trafficking and metabolism-related differential GO terms. CK: control group; TM: exposed group.

To explore the biological responses of *E. carinicauda’s* hepatopancreas after being exposed to *A. pacificum*, a GO enrichment analysis was performed, with the focus being on biological processes, cellular components, and molecular function (Figure 4A). Within the biological process category, 21 level 2 GO terms were enriched following exposure, and these included regulation of the biological process, biological regulation, metabolic process, and cellular process. Similarly, DEGs were primarily associated with protein-containing complex and cellular anatomical entities in the cellular component category, while for molecular function, the top two enriched level 2 GO terms were binding and catalytic activity. Further analysis revealed significant enrichment in 10 GO terms related to protein trafficking and metabolism (Figure 4B), including lipid transporter activity, xenobiotic transmembrane transporter activity, isoprenoid binding, monocarboxylic acid transmembrane transporter activity, xenobiotic transport, transition metal ion transmembrane transporter activity, late endosome, ABC-type transporter activity, neutral amino acid transport, and phosphate ion transport. Finally, the results of the GSEA revealed significant downregulation of the genes involved in these processes in the exposed group (Figure 4C).

### 2.5. KEGG Enrichment Analysis

**Figure 5:**
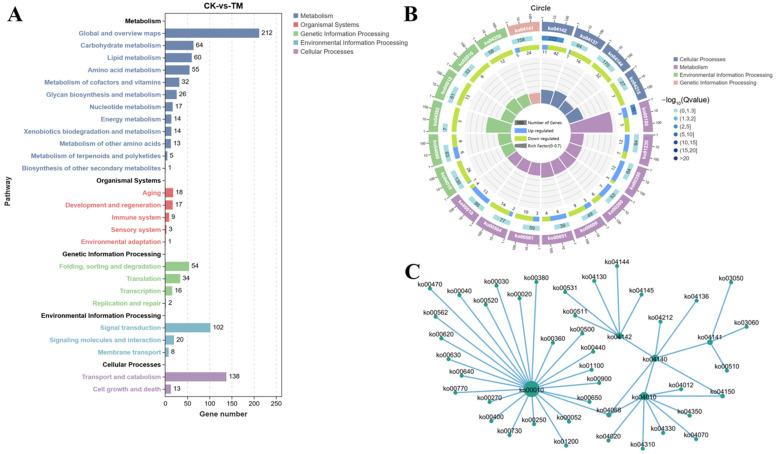
KEGG enrichment analysis of DEGs in the hepatopancreas of *E. carinicauda* from the exposed and control groups. (**A**) DEGs mapped to KEGG Class A and Class B categories. (**B**) Significantly enriched KEGG pathways. (**C**) Network plot illustrating interactions among key pathways. CK: Control group; TM: Exposed group.

A KEGG enrichment analysis identified 1007 DEGs mapped to 5 KEGG Class A and 26 KEGG Class B categories (Figure 5A). Notably, exposure to *A. pacificum* significantly impacted 20 pathways, including lysosome, endocytosis, metabolic pathways, and the MAPK signaling pathway (Figure 5B). Furthermore, a network plot highlighted the key pathways altered by *A. pacificum* exposure (Figure 5C), and these included protein processing in the ER (ko04141), lysosome (ko04142), glycolysis/gluconeogenesis (ko00010) and the MAPK signaling pathway (ko04010).

### 2.6. Differentially Expressed Genes Involved in Different Functions

Table 1 summarizes the genes involved in key metabolic and functional pathways in the hepatopancreas, such as protein processing, mitochondrial function, sphingolipid metabolism, glycerophospholipid metabolism, and glycolysis/gluconeogenesis. Among the 18 genes associated with protein processing, only sec61 translocon subunit beta (*SEC61B*); lectin, mannose binding 1 (*LMAN1*); and crystallin, alpha B (*CRYAB*) were upregulated. In contrast, 11 genes related to mitochondrial function in the hepatopancreas were significantly downregulated. Furthermore, six genes were linked to sphingolipid metabolism in the hepatopancreas, with all but galactose-3-O-sulfotransferase 1 (*GAL3ST1*) showing downregulation, while among the 11 genes associated with glycerophospholipid metabolism, all, except for n-myristoyltransferase 2 (*NMT2*), were downregulated. Finally, nine genes related to glycolysis/gluconeogenesis were identified, and these included triosephosphate isomerase 1a (*tpi1a*), aldehyde dehydrogenase 3 family member A2 (*Aldh3a2*), enolase (*Eno*), fructose-1,6-bisphosphatase 1 (*FBP1*), alcohol dehydrogenase 5 (Class III), chi polypeptide (*ADH5*), phosphoglycerate kinase (*Pgk*), lactate dehydrogenase (LDH), aldehyde dehydrogenase 3 family member a1 (*Aldh3a1*), and phosphoenolpyruvate carboxykinase 2, mitochondrial (*PCK2*).

### 2.7. Validation of Gene Expression Through Quantitative Reverse-Transcription PCR (qRT-PCR)

**Figure 6:**
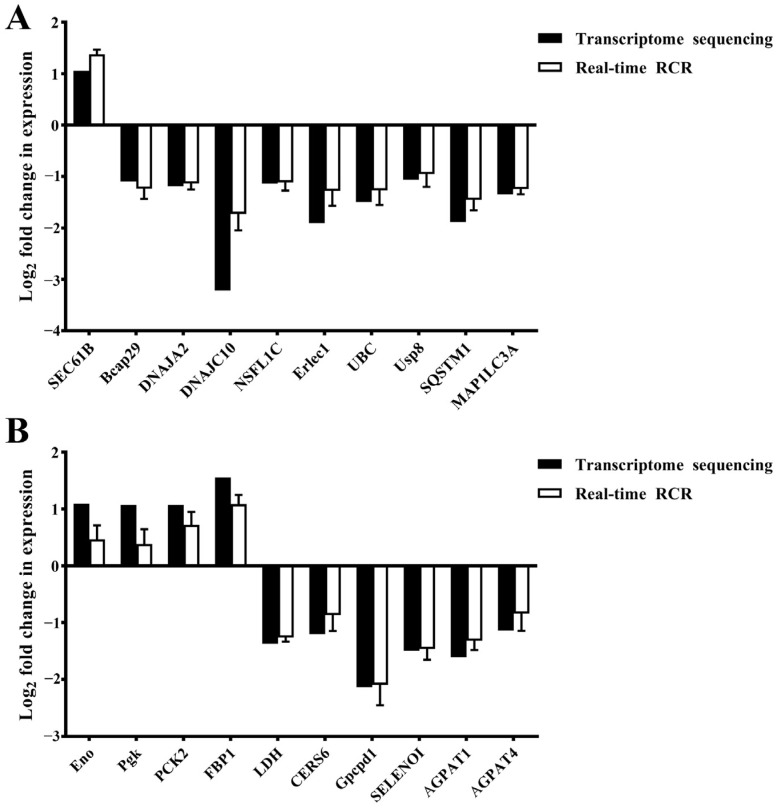
Validation of the expression levels of 20 DEGs from the transcriptome using qRT-PCR (**A**,**B**).

To validate the RNA-seq results, 20 DEGs involved in protein processing, mitochondrial function, sphingolipid metabolism, glycerophospholipid metabolism, and glycolysis/gluconeogenesis were selected. The expression levels of these genes were then analyzed by qRT-PCR, with the results being consistent with those observed in the RNA-seq analysis (Figure 6).

## 3. Discussion

The dinoflagellate *A*. *pacificum*, a typical HAB species, leads to significant environmental issues, substantial economic losses in the aquaculture industry, and potential threats to human health [15,26]. Crustaceans are particularly sensitive to environmental changes, thus making shrimp ponds effective early warning signs for HABs. However, limited information is available regarding the detrimental effects of *A. pacificum* on *E. carinicauda*. Therefore, the current work investigated the adverse effects of *A. pacificum* on the hepatopancreas of *E. carinicauda* by examining the organ’s histological structure, antioxidant enzyme activity, and transcriptomic profile.

The hepatopancreas is a vital organ in shrimp as it serves as the primary site for metabolic processes and detoxification while being also a potential target for diseases [27]. Research has shown that environmental stress can induce oxidative damage in the hepatopancreas, thereby increasing its susceptibility to apoptosis [28]. For instance, acute nitrite stress in *Litopenaeus vannamei* caused abnormal star-shaped tubular structures in its hepatopancreas, with hepatic tubules also undergoing atrophy and detachment from the basement membrane [29]. Similarly, a 21-day exposure to nanopolystyrene and phosphorus oxide in *Eriocheir sinensis* compromised the mechanical barrier of the hepatopancreas [30]. In the exposed group, *E. carinicauda* exhibited pathological hepatopancreatic damage, including lumen expansion and the detachment of the epithelial cells from the basement membrane. It was, therefore, hypothesized that *A. pacificum* can disrupt physiological homeostasis in the hepatopancreas of *E. carinicauda*, with the observed tissue damage likely due to its role as a primary target organ for toxic dinoflagellates.

Environmental stressors, including toxic contaminants, are well-documented inducers of oxidative stress in aquatic organisms [31]. Antioxidant enzymes, such as SOD, CAT, and glutathione-dependent ones, play a crucial role in mitigating oxidative damage caused by reactive oxygen species (ROS). Thus, under biotic and abiotic stress conditions, the overexpression of these enzymes often serves as a defense mechanism [32]. However, this study showed that the activities of SOD, CAT, and GSH in the hepatopancreas of *E. carinicauda* were significantly reduced following exposure to *A. pacificum*. Similar findings, notably a significant decrease in SOD and CAT activities, were reported in *L. vannamei* which had been exposed to nitrite and alkaline environmental stress [28,33]. In the exposed group, the observed reduction in antioxidant enzyme activity could be attributed to the inhibitory effects of high concentrations of toxic *A. pacificum* on enzymatic function. This inhibition subsequently compromises the antioxidant defense system, resulting in increased levels of lipid peroxidation. The damage to hepatopancreatic tissues also likely disrupts cellular defense mechanisms, including antioxidant pathways [34].

### 3.1. Key Genes Associated with Protein Processing and Mitochondrial Function

Environmental stressors are known to impair the structure and function of protein-folding enzymes, thus reducing their capacity to achieve the proper folding of newly synthesized proteins. This dysfunction also results in the accumulation of misfolded proteins within the endoplasmic reticulum [35]. In this study, significant alterations were observed in the genes associated with protein processing. Among these, dnaJ heat shock protein family (*Hsp40*) member A2 (*DNAJA2*) and dnaJ heat shock protein family (*Hsp40*) member C10 (*DNAJC10*) are well-characterized molecular chaperones that play crucial roles in protein folding and serve as biomarkers of environmental stress sensitivity [36]. Specifically, DNAJA2 is involved in protein folding and the mitochondrial import of proteins, thus ensuring the proper folding of proteins and preventing the accumulation of misfolded variants. Conversely, DNAJC10 is a part of the endoplasmic reticulum-associated degradation complex which identifies and degrades misfolded proteins, thereby reducing the formation of improper disulfide bonds in misfolded glycoproteins. Studies on *Procambarus clarkii* have shown that high-temperature stress can downregulate genes involved in protein folding and processing in the hepatopancreas, thus leading to the accumulation of misfolded proteins [37]. In this study, the expression levels of *DNAJA2* and *DNAJC10* were significantly downregulated in the hepatopancreas of *E. carinicauda*. In this context, it was hypothesized that exposure to the toxic dinoflagellates *A. pacificum* may lead to the accumulation of misfolded proteins, disruption of the regulatory mechanisms of *E. carinicauda*, damage to hepatopancreatic tissues, and impaired physiological functions of hepatopancreatic cells [38].

Environmental stress often triggers the accumulation of ROS in the hepatopancreas, with this process potentially leading to mitochondrial dysfunction [39]. The ubiquitin c (*UBC*) modifies mitochondrial proteins through ubiquitination, thereby playing a critical role in mitochondrial quality control and mitophagy [40]. Similarly, ubiquitin-specific protease 8 (*USP8*) can remove K6-linked ubiquitin chains formed by Parkin and regulate the subsequent mitophagy process. Without *USP8*, aggregated ubiquitin chains hinder Parkin’s interactions with other receptors, such as sequestosome 1 (*SQSTM1*) and microtubule-associated protein 1 light chain 3 alpha (*MAP1LC3A*), thereby disrupting mitochondrial autophagy [41]. Previous studies have highlighted that an imbalance in mitochondrial homeostasis can exacerbate oxidative stress and apoptosis while disrupting overall organismal homeostasis [42]. For instance, in *L. vannamei*, reduced *SQSTM1* levels under low-temperature stress can intensify hepatopancreas damage [43]. Additionally, in mitten crabs, increased MAP1LC3A protein levels have been observed following exposure to microcystin-LR, hence underscoring the crucial role of mitochondrial regulation in stress responses [44]. In this study, the expression levels of *UBC*, *USP8*, *SQSTM1,* and *MAP1LC3A*, which are involved in mitochondrial function, were all significantly downregulated in the exposed group. It was speculated that oxidative stress induced by *A. pacificum* could compromise the cells’ ability to effectively manage environmental stressors, leading to hepatopancreatic damage. Impaired mitochondrial function may also hinder cellular adaptation to changes in the external environment, potentially activating apoptotic pathways and increasing cell death. This cascade of events may adversely impact the growth and development of shrimp. Furthermore, damage to hepatopancreatic tissues can disrupt regulatory mechanisms within this organ, thereby affecting its normal physiological functions [45].

### 3.2. Key Genes Related to Energy Metabolism

Energy is essential for sustaining normal life activities, and in the case of crustaceans, regulating energy metabolism enables their adaptation to environmental changes [46]. In this context, the hepatopancreas is a crucial organ for energy storage and metabolism, providing the necessary energy for stress responses [47]. Glycolysis, a vital pathway for carbohydrate metabolism, is widespread among animals, with its key purpose being the production of ATP under hypoxic conditions to fulfill the energy demands of cells devoid of mitochondria or during environmental stress [48]. Among the key components in this process, *Pgk* is the first significant metabolic enzyme responsible for ATP generation during glycolysis. A transcriptome analysis undertaken in this study revealed that the expression levels of *tpi1a*, *Eno*, and *Pgk*, all of which are involved in the glycolytic pathway, were upregulated, while *LDH* expression was downregulated. LDH is a critical enzyme in the glycolytic pathway as it facilitates the interconversion between pyruvate and lactate. Previous studies have demonstrated that the upregulation of LDH activated the glycolysis pathway under environmental stress [49]. Conversely, in this study, the downregulation of *LDH* in the hepatopancreas of the exposed group could be attributed to a cellular shift towards the gluconeogenesis pathway as a means of adapting to environmental changes. This adjustment to maintain the balance of energy and metabolites could also be linked to structural damages and impaired physiological functions observed in the hepatopancreatic cells after exposure to *A. pacificum*. Gluconeogenesis is another critical pathway that generates ATP by converting non-carbohydrate substances into glucose under aerobic conditions [50]. PCK2, a key enzyme in this process, serves as a central molecule linking the TCA cycle, glycolysis, and gluconeogenesis [51]. Meanwhile, fructose-1,6-bisphosphatase 1 (*FBP1*) acts as a crucial regulator of the gluconeogenesis pathway, facilitating the hydrolysis of fructose 1,6-bisphosphate into fructose 6-phosphate and inorganic phosphate, thereby controlling the rate of gluconeogenesis [52]. The current findings suggested that the downregulation of *PCK2* could be attributed to the disruption of hepatopancreatic energy metabolism caused by exposure to *A. pacificum*, while *FBP1* upregulation may result from the activation of the gluconeogenesis pathway during glycolysis to adapt to environmental changes. Previous studies suggested that low temperature stress may downregulate *FBP1* expression in the hepatopancreas of *L. vannamei*, thereby inhibiting the gluconeogenesis pathway while activating the glycolysis pathway to meet energy demands under such conditions [53]. Additionally, research indicated that the increased expression of the genes related to energy metabolism in the shrimp hepatopancreas was essential for maintaining normal physiological functions and enhancing tolerance to environmental changes [54].

This study observed a significant downregulation of ceramide synthase 6 (*CERS6*), 1-acylglycerol-3-phosphate o-acyltransferase 1 (*AGPAT1*), and 1-acylglycerol-3-phosphate o-acyltransferase 4 (*AGPAT4*), the genes involved in sphingolipid and glycerophospholipid metabolism. Sphingolipid metabolism, a crucial component of lipid metabolism, plays a key role in maintaining cellular homeostasis and responding to environmental stressors [55]. The hepatopancreas, the site of ceramide production, is rich in sphingolipids and highly sensitive to environmental changes. For instance, studies have shown that a reduction in *CERS6* expression directly reduced the activity of the encoded enzyme, thereby diminishing the synthesis of specific chain-length ceramides [56]. Pauletto et al. (2018) further found that after aeration stress, *CERS6* expression in *Pecten maximus* larvae was lower than that of the control group, suggesting that stress conditions may impair the efficiency of lipid metabolism and lead to delayed growth [57]. Animal cell membranes are also rich in glycerophospholipids which are critical to their structure and function [58], with their metabolism being closely related to biological functions and oxidative stress [59]. Furthermore, under oxidative stress, glycerophospholipid metabolism helps to maintain the cell membrane’s stability, and this process is associated with lipid peroxidation and stress mechanisms [60]. Notably, phosphatidic acid (PA), produced by AGPATs, serves as a key metabolite in glycerophospholipid metabolism, with AGPATs being central to this process [61]. The hepatopancreas is vital for crustacean functions, such as lipid metabolism which is essential for growth [62]. The current study found that *CERS6*, *AGPAT1* and *AGPAT4* were all downregulated in the hepatopancreas of *E. carinicauda* following exposure to the toxic dinoflagellates *A. pacificum*. In particular, the downregulation of *CERS6* is speculated to be due to an imbalance in sphingolipid levels which disrupts the equilibrium between cholesterol and sphingolipids in the plasma membrane. Such disruption may further alter nutrient availability, thereby affecting the overall cellular homeostasis and function, potentially leading to metabolic disorders. Concurrently, the downregulation of *AGPAT1* and *AGPAT4* expression was expected for lowering the synthesis of PA. This may impact phospholipid synthesis and lead to changes in intracellular lipid storage, which may further affect cell growth and survival. Collectively, these findings suggest that the toxin-producing dinoflagellates *A. pacificum* may induce energy metabolism disorders in *E. carinicauda*, thereby interfering with their normal growth and development. This process may also be associated with the production of ROS [63].

## 4. Materials and Methods

### 4.1. Shrimp Selection and Culture Conditions

Experimental *E. carinicauda*, with an average weight and body length of 0.8 ± 0.02 g and 5.14 ± 0.38 cm, respectively, were selected from a healthy population sourced from a farm in Lianyungang city, Jiangsu Province, China. Before the experiment, the shrimp were allowed to acclimatize for one week in 40 L plastic tanks with continuous aeration, and during this period, one-third to one-half of the water was replaced daily using a drip method. In addition, the culture conditions were maintained at a pH, salinity, and temperature of 8.0–8.2, 24–26, and 25 ± 1 °C, respectively. The shrimp were fed a commercial diet at regular intervals each day, and the dead ones or waste materials were promptly removed.

### 4.2. Alexandrium Pacificum Culture

*A. pacificum* was cultured in 5 L Erlenmeyer flasks containing f/2 medium without silicate (Si). Batch cultivation was then conducted at a temperature of 20 ± 1 °C and under a light intensity of 60 ± 10 μEm^−2^ s^−1^ with a 14 h:10 h light-to-dark photoperiod. The culture medium was prepared using natural seawater (salinity of 25) which was collected from the Lianyungang area, filtered through a 0.5 μm membrane, and eventually sterilized under high temperature and pressure before use. Prior to the experiment, the algae were fixed with Lugol’s fixative and counted using a 1 mL plankton counting chamber.

### 4.3. Experimental Design and Sample Collection

After one week of acclimation, the shrimp were randomly assigned to one of the following two groups: one exposed to *A. pacificum* at a concentration of 1.0 × 10^4^ cells/mL (exposed group) and control group. Each group consisted of three replicates, with 40 *E. carinicauda* individuals randomly assigned per replicate. Every 12 h, the algal cells were fixed using Lugol’s fixative and counted under a microscope with a 1 mL plankton counting chamber. In addition, the culture medium was replaced every 24 h to maintain the concentration of *A. pacificum* at the designated level. The experiment lasted 7 days, during which the concentration in the exposed group was consistently maintained at 1.0 × 10^4^ cells/mL. At the end of the experimental period, hepatopancreas tissues were collected from nine randomly selected *E. carinicauda* individuals from each group. These tissues were then homogenized with 9 volumes (*w*/*v*) of physiological saline at 4 °C for the subsequent biochemical analysis. Similarly, hepatopancreas tissues obtained from nine randomly selected shrimp from each group were used for the transcriptome analysis. In this case, tissues from three shrimp were pooled into a single sample, with all the samples eventually stored at −80 °C for further analyses.

### 4.4. Histopathological Analyses

After the seven-day exposure, hepatopancreas tissues were collected from nine *E. carinicauda* individuals from each group. These tissue samples were preserved in paraformaldehyde for 24 h, after which dehydration was performed using 70% ethanol to remove residual water. This was followed by xylene treatment to facilitate paraffin infiltration, with the tissues eventually embedded in melted paraffin for thorough impregnation. The paraffin-embedded samples were then sliced into 4–6 μm thick sections using a microtome and mounted onto glass slides for drying. These sections were subsequently stained with hematoxylin and eosin (H&E) in order to visualize tissue alterations under an optical microscope.

### 4.5. Measurement of Hepatopancreas Antioxidant Parameters

Hepatopancreas tissues were accurately weighed and homogenized (1:9, *v*/*w*) in 0.85% ice-cold saline solution [64] prior to a 10 min centrifugation at 3000 rpm and 4 °C. The resulting supernatant was then stored at −80 °C until required for the subsequent determination of the total protein content. Additionally, malondialdehyde (MDA, A003-1-2, TBA) and glutathione (GSH, A006-2-1) levels as well as the activities of catalase (CAT, A007-1-1, visible light) and superoxide dismutase (SOD, A001-3-2, WST-1) in the hepatopancreas were determined, with commercial kits (Jiancheng Bioengineering Institute, Nanjing, China) used to measure all the enzyme activities.

### 4.6. Transcriptome Sequencing in Hepatopancreas

A UNlQ-10 Column Trizol Total RNA Isolation Kit was used to extract total RNA from the hepatopancreas tissues of *E. carinicauda* individuals of the control and exposed groups. The RNA quality was then assessed before synthesizing cDNA through reverse transcription. Following the purification of the double-stranded cDNA, a library was constructed prior to sequencing on an Illumina Nova 6000 system (Gene Denovo Biotechnology Co., Ltd., Guangzhou, China). Quality control of the raw sequences was performed using FASTP (version 0.18.0) to remove low-quality reads [65]. Clean reads were aligned to the reference genome assembly [66] using the HISAT v2.0 program. The resulting high-quality data were then subjected to a principal component analysis (PCA) to evaluate the relationship among the samples. Additionally, the DESeq2 software (version 1.20.0) was used to identify differentially expressed genes (DEGs) between the exposed and control groups, with an absolute log2 fold change (log2 FC) of ≥2 and a false discovery rate (FDR) of <0.05 selected as screening thresholds [67]. Biological functions and key signaling pathways were also explored through Gene Ontology (GO) (http://www.geneontology.org/, accessed on 23 October 2024) and Kyoto Encyclopedia of Genes and Genomes (KEGG) enrichment analyses (http://www.kegg.jp/, accessed on 23 October 2024) before using the Gene Set Enrichment Analysis (GSEA) (version 2.2.4) for identifying distinct GO terms and pathways between the exposed and control groups. In this case, an FDR of <0.05, a *p*-value of <0.05, and a normalized enrichment score (NES) of >1 were selected as the threshold for significance [68].

### 4.7. Quantitative Reverse-Transcription PCR (qRT-PCR)

The qRT-PCR analysis was performed to validate the accuracy of the transcriptome data. For this purpose, RNA was extracted and subsequently reverse transcribed into cDNA using the UNlQ-10 column-based Trizol Total RNA Extraction Kit (Shenggong Biological Engineering Co., Ltd., Shanghai, China) and the Trans Script^®^ One-Step gDNA Removal and cDNA Synthesis Super Mix (TransGen Biotech Co., Ltd., Beijing, China), respectively. This was followed by qPCR, performed on a Multiwell Plate 96 reaction system (LightCycler^®^ 96, Roche, Basel, Switzerland) using the TransGen Biotech Perfect Start^®^ Green qPCR Super Mix kit (TransGen Biotech Co., Ltd., Beijing, China) according to the provided instructions. The 2^−ΔΔCT^ method was eventually used to assess the relative expression levels of each target gene, with 18S selected as the reference [69]. All the primers used in this study were designed using Primer Premier 5.0 and are listed in Table S2.

### 4.8. Statistical Analysis

The results were statistically analyzed in the SPSS 27.0 software using one-way analysis of variance (ANOVA) and Duncan’s multiple range test to determine their significance at *p* < 0.05.

## 5. Conclusions

In this study, the toxicological effects of *A. pacificum* on the hepatopancreas of *E. carinicauda* were thoroughly examined through histological analysis, antioxidant enzyme activity assays, and transcriptomic profiling. The findings indicated that exposure to *A. pacificum* resulted in significant histological damage along with significant changes in antioxidant parameters, such as SOD, CAT, GSH, and MDA, which were indicative of hepatic dysfunctions. The transcriptomic analyses further identified 2398 differentially expressed genes that were primarily involved in protein processing, mitophagy, mitochondrial function, sphingolipid metabolism, and glycerophospholipid metabolism. From the results, it was hypothesized that *A. pacificum* likely exerted its toxicological effects by disturbing hepatic protein processing, mitophagy, mitochondrial function, and energy metabolism. Altogether, these findings offer new insights into the adverse impact of harmful toxin-producing dinoflagellates on aquatic animals while also assisting in the ecological risk assessment of harmful algal blooms in aquaculture.

## Figures and Tables

**Table 1:** Summary of DEGs in significantly enriched pathways in the hepatopancreas of *E. carinicauda*.

KEGG Pathway	Gene ID	Gene Name	Regulation	Log_2_FC
Protein processing	Unigene0004773	SEC61 translocon subunit beta (*SEC61B*)	↑	1.0581
	Unigene0035173	Lectin, Mannose Binding 1 (*LMAN1*)	↑	1.2096
	Unigene0011351	DnaJ heat shock protein family (Hsp40) member C3 (*DNAJC3*)	↓	−1.3528
	Unigene0018843	Cullin 1 (*CUL1*)	↓	−1.6686
	Unigene0019622	DnaJ heat shock protein family (Hsp40) member C5 (*DNAJC 5*)	↓	−2.2786
	Unigene0021323	ER Degradation Enhancing Alpha-Mannosidase Like Protein 3 (*edem3*)	↓	−1.6412
	Unigene0017803	DnaJ heat shock protein family (Hsp40) member C10 (*DNAJC10*)	↓	−3.2146
	Unigene0006430	B Cell Receptor-Associated Protein 29 (*Bcap29*)	↓	−1.0944
	Unigene0020150	ubiquitination factor E4B (*UBE4B*)	↓	−1.5901
	Unigene0006758	DnaJ heat shock protein family (Hsp40) member A2 (*DNAJA2*)	↓	−1.1873
	Unigene0006469	crystallin, alpha B (*CRYAB*)	↑	1.8274
	Unigene0024253	Thioredoxin Domain Containing 5 (*TXNDC5*)	↓	−1.1330
	Unigene0042968	Membrane-bound transcription factor peptidase, site 1 (*MBTPS1*)	↓	−1.5759
	Unigene0018951	NSFL1 cofactor (*NSFL1C*)	↓	−1.1385
	Unigene0039288	Endoplasmic Reticulum Lectin 1 (*Erlec1*)	↓	−1.9039
	Unigene0008319	lethal (2) essential for life [*l(2)efl*]	↓	−2.1341
	Unigene0014712	Membrane-associated ring-CH-type finger 6 (*MARCHF6*)	↓	−1.0324
	Unigene0017804	Ubiquitin Recognition Factor In ER-Associated Degradation 1 (*Ufd1*)	↓	−1.2287
Mitophagy	Unigene0041625	Sequestosome 1 (*SQSTM1*)	↓	−1.8847
	Unigene0033333	Ubiquitin C (*UBC*)	↓	−1.4974
	Unigene0038070	Autophagy-related protein 9A (*ATG9A*)	↓	−2.2367
	Unigene0034577	Muscle RAS Oncogene Homolog (*MRAS*)	↓	−1.5686
	Unigene0009963	Jun Proto-Oncogene, AP-1 Transcription Factor Subunit (*JUN*)	↓	−1.3923
	Unigene0038584	GABA type A receptor-associated protein (*GABARAP*)	↓	−1.3066
	Unigene0019289	TANK binding kinase 1 (*tbk1*)	↓	−1.5371
	Unigene0003988	Microtubule-associated protein 1 light chain 3 alpha (*MAP1LC3A*)	↓	−1.3475
	Unigene0007421	Ras oncogene at 85D (*Ras85D*)	↓	−1.1482
	Unigene0043075	Ubiquitin Specific Peptidase 8 (*Usp8*)	↓	−1.0614
	Unigene0032676	TBC1 Domain Family Member 15 (*TBC1D15*)	↓	−1.3495
Sphingolipid metabolism	Unigene0039670	Sphingomyelin Phosphodiesterase 1 (*SMPD1*)	↓	−1.2652
	Unigene0041427	UDP-glucose ceramide glucosyltransferase (*ugcg*)	↓	−1.9397
	Unigene0000281	sphingosine-1-phosphate lyase (*Sply*)	↓	−1.3089
	Unigene0020466	galactose-3-O-sulfotransferase 1 (*GAL3ST1*)	↑	1.0295
	Unigene0032984	Ceramide Synthase 6 (*CERS6*)	↓	−1.2039
	Unigene0016910	Burrows-Wheeler Aligner (*bwa*)	↓	−2.1083
Glycerophospholipid metabolism	Unigene0003027	N-myristoyltransferase 2 (*NMT2*)	↑	1.7817
	Unigene0001693	lipin 2 (*LPIN2*)	↓	−2.5149
	Unigene0005731	Phospholipase D beta 1 (*PLDBETA1*)	↓	−1.7478
	Unigene0040271	1-acylglycerol-3-phosphate O-acyltransferase 4 (*AGPAT4*)	↓	−1.1435
	Unigene0033720	glycerol-3-phosphate acyltransferase 4 (*Gpat4*)	↓	−1.4497
	Unigene0039712	Glycerophosphocholine Phosphodiesterase 1 (*Gpcpd1*)	↓	−2.1396
	Unigene0019010	glycerol-3-phosphate dehydrogenase 2 (*Gpd2*)	↓	−2.2879
	Unigene0019229	Glycerol-3-phosphate dehydrogenase 1 (*Gpdh1*)	↓	−1.1828
	Unigene0012708	lysophosphatidylglycerol acyltransferase 1 (*LPGAT1*)	↓	−1.5238
	Unigene0001374	1-Acylglycerol-3-Phosphate O-Acyltransferase 1 (*AGPAT1*)	↓	−1.6105
	Unigene0008571	Selenoprotein I (*SELENOI*)	↓	−1.4981
Glycolysis/Gluconeogenesis	Unigene0001370	Triosephosphate Isomerase 1a (*tpi1a*)	↑	1.1835
	Unigene0004971	Aldehyde Dehydrogenase 3 Family Member A2 (*Aldh3a2*)	↓	−1.8675
	Unigene0010840	Enolase (*Eno*)	↑	1.0917
	Unigene0039462	Fructose-1,6-bisphosphatase 1 (*FBP1*)	↑	1.5572
	Unigene0033523	Alcohol Dehydrogenase 5 (Class III), Chi Polypeptide (*ADH5*)	↑	1.4259
	Unigene0013435	Phosphoglycerate Kinase (*Pgk*)	↑	1.0731
	Unigene0000916	Lactate Dehydrogenase (*LDH*)	↓	−1.3764
	Unigene0037089	Aldehyde Dehydrogenase 3 Family Member A1 (*Aldh3a1*)	↓	−1.4323
	Unigene0002179	Phosphoenolpyruvate Carboxykinase 2, Mitochondrial (*PCK2*)	↓	−1.6471

Note: “↓” indicate downregulation; “↑” indicate upregulation.

## Data Availability

In this study, all the data generated are included in this article and Supplementary Materials. Further enquiries can be directed to the corresponding author.

## Supplementary Materials

The following supporting information can be downloaded at https://www.mdpi.com/article/10.3390/ijms26041605/s1.
