# Supplementary material for: Effects of Alexandrium pacificum Exposure on Exopalaemon carinicauda: Hepatopancreas Histology, Antioxidant Enzyme Activity, and Transcriptome Analysis
Source: Int J Mol Sci. 2025 Feb 13;26(4):1605. doi: 10.3390/ijms26041605 (PMC11855214; doi:10.3390/ijms26041605)
Supplement: Supplementary file 1 [file ijms-26-01605-s001.zip › ijms-3463717-supplementary.pdf]

Table S1. Valid data used in transcriptome analysis.

Samples	Raw data（bp）	Clean data（bp）	Q20 (%)	Q30 (%)	GC (%)
CK-1	7,712,067,300	7,620,206,372	96.81	91.18	44.67
CK-2	7,186,239,900	7,094,864,499	96.87	91.28	44.64
CK-3	7,285,840,500	7,186,089,918	96.94	91.45	44.64
TM-1	7,847,334,000	7,749,082,188	96.99	91.53	45.31
TM-2	6,746,112,300	6,677,242,998	98.70	95.89	45.15
TM-3	6,378,040,500	6,322,223,082	98.61	95.59	45.20

Note: Q20 and Q30, the base quality score (Q score) was no less than 20 and 30, respectively, in clean reads; GC, GC content in clean reads; CK: Control group TM: Exposed group.

Table S2. Primers used for qRT-PCR in the study.

Gene Name	Primer sequence (5′-3′)	Products size(bp)	Melting temperature (℃)
B Cell Receptor Associated Protein 29 (Bcap29)	F: TCTGGATTTTCCCTCTTTC<br>R: TGCTGATCTTCCTCTTTTC	173	52<br>52
DnaJ heat shock protein family (Hsp40) member A2 (DNAJA2)	F: GGGGGTATGAGGGGACAGA<br>R: TTGAAGAGCACCAGCAGGA	171	59<br>58
SEC61 translocon subunit beta (SEC61B)	F: GCCTCTCCTAGTTCAACCA<br>R: TGTAAAACCTCCACATCCC	175	56<br>54
Endoplasmic Reticulum Lectin 1 (Erlec1)	F: GATATACCACAGAACGACC<br>R: TCACCACTTAACACAGCAC	125	52<br>55
DnaJ Heat Shock Protein Family (Hsp40) Member C10 (DNAJC10)	F: ATCGCAAGTAAACCACAAG<br>R: GCCATACACAGCACAGTCA	159	53<br>57
NSFL1 cofactor (NSFL1C)	F: TTTTTACGCAGGTGGCTCT<br>R: TTGGTTTGGACTTTTTCCC	149	57<br>53
Ubiquitin C (UBC)	F: TCACCAGCCTAAAGAAGCA<br>R: ACCAACACCCAGACAAAAA	348	56<br>55
Ubiquitin Specific Peptidase 8 (Usp8)	F: GTGCCACTTTACCATCCAT<br>R: GTGTTGCCATTTTCCCATT	199	55<br>54
sequestosome 1 (SQSTM1)	F: CGTGGATGGAGAGGATGGT<br>R: GCTGCTGCTGATTGGAAAA	109	58<br>56
microtubule associated protein 1 light chain 3 alpha (MAP1LC3A)	F: AACAAATTCGAGAACAGCA<br>R: ATAAGCAAGAAGAAAGCCT	187	53<br>51
Ceramide Synthase 6 (CERS6)	F: ATGCTCCAACTATTGTCCC<br>R: TTTTCTCTCGTCGTCTTCC	197	54<br>54
Enolase (Eno)	F: TTACCCGAAAAAAAGCAGA<br>R: ACCAGTGAGGGATTCCAAC	129	52<br>56
Phosphoenolpyruvate Carboxykinase 2, Mitochondrial (PCK2)	F: GCTTTCCCTTCTGCCTGTG<br>R: TAAAATTCCATCCTCGTCG	123	58<br>53
Phosphoglycerate Kinase (Pgk)	F: ATCCAGAGAGACCCTTCCT<br>R: TTGCTCCATCCTCATCATA	183	56<br>52
Lactate Dehydrogenase (LDH)	F: ATGGTGGGGCTGTTCAGGA<br>R: CAAGGCAACGGTGGGATTC	191	60<br>59
Fructose-1,6-bisphosphatase 1 (FBP1)	F: AAGTAGGCGTGGCTGTAGT<br>R: GTCAGTGTCATGGAATCGG	103	58<br>55
Selenoprotein I (SELENOI)	F: ATAGATGGGAAACAAGCAC<br>R: ACACAGATAGGAGGCAAAA	115	52<br>53.
Glycerophosphocholine Phosphodiesterase 1 (Gpcpd1)	F: TTCGGCAAAACATATTCCC<br>R: ACCCACTTCCAGTCCCTTC	133	53<br>58
1-acylglycerol-3-phosphate O-acyltransferase 4 (AGPAT4)	F: GCTCTCCATCTACTGTTGT<br>R: GACTCTTGTTGAAGGGTCT	103	53<br>54
1-Acylglycerol-3-Phosphate O-Acyltransferase 1 (AGPAT1)	F: CCTGAGGGTACAAGAAACA<br>R: TAAACAACTGGCAAGATGG	176	53<br>53
18S (Internal reference)	F: TATACGCTAGTGGAGCTGGAA<br>R: GGGGAGGTAGTGACGAAAAAT	147	58<br>57
